# 

**Published:** 1899-06-24

**Authors:** 


**Notices of Births, Deaths, and Marriages** are inserted in this column at a charge of 2s. 6d. for an announcement not exceeding four lines.

### NOTICE TO CORRESPONDENTS.

All MS., Letters, Books for Review, and other matters intended for the Editor should be addressed The Editor, "The Hospital," The Hospital Building, 28 & 29, Southampton Street, Strand, W.C.

The Editor cannot undertake to return rejected MS., even when accompanied by stamped directed envelope.

**Notice to Advertisers and Subscribers.**

All Advertisements, Orders for Copies of The Hospital, and Business Communications should be addressed to **THE MANAGER (Not to the Editor),** The Hospital, The Hospital Building, 28 & 29, Southampton Street, Strand, London, W.C.

The Cost of Subscription, post free, is as follows:—Per week, 2½d.; per quarter, 2s. 8d.; per half-year, 5s. 3d.; per annum, 10s. 6d. Foreign:—Per annum, 15s. 2d., payable, in all cases, in advance.

**NOTICE.—Vol. XXV. of "The Hospital" (October, 1898, to March, 1899), in handsome cloth binding, is now on sale at the Office of this Journal, price 7s. 6d. Cases for binding the Numbers contained in this Volume 1s. 6d. Index to Vol. XXV., 2½d., post free.**

**The Monthly Part for July will be ready on July 1st. Price 6d., by post 8d.**

### BOOKS RECEIVED.

Baillière et Cie, Paris.

"Dictionaire de Physiologie." Par Charles Richet, Premier Fascicule du Tome IV.

G. Van Abbott and Sons.

"Cookery Receipts for Diabetics."

Charles Griffin and Co.

"Year Book of Scientific and Learned Societies."

John Bale, Sons, and Danielsson.

"Selby." By Eppie Frazer.

West, Newman, and Co.

"Archives of Surgery." April, 1899. By Jonathan Hutchinson, LL.D., F.R.S.

George Allen.

"Some Thoughts about Nursing" and "The Care of the Dying." By Oswald Browne, M.A., M.D., F.R.C.P.

Ward, Lock, and Co.

"Master and Servant."

Grant and Co.

"The Code for Day Schools, 1899-1900." Edited by Herbert Cornish.

Henry J. Glaisher.

"The Treatment of Hæmorrhoids." By Dudley Wright, F.R.C.S.
"Lord Lister and Surgery." By Robert Turner, M.A., M.D.

H. H. G. Grattan.

"The Pharmacopœia of Guy's Hospital." Compiled by a Committee of the Staff.

The Church Shop, Commercial Road.

"Through the Long Night Watch."

The F. A. Davis Co.

"A Text Book of Practical Obstetrics." By Egbert H. Grandin, M.D., and George W. Jarman, M.D.
"The Anatomy of the Central Nervous System of Man." By Ludwig Edinger, M.D. Translated by Winfield S. Hall, M.D., Philo Leon Holland, M.D., and Edward P. Carlton, B.D.
"An Epitome of the History of Medicine." By Roswell Park, A.M., M.D.

Baillière, Tindall, and Cox.

"Essentials of Modern Treatment of Disease." By K. M. Nadkarni.
"Aids to the Treatment of Diseases of Children." By John McCaw, M.D.

Her Majesty's Stationery Office.

"Annual Report of Proceedings under the Diseases of Animals Acts for the Year 1898."

Scientific Press.

"Pritchard's London and Londoners, 1899." Edited by Rosalind Pritchard.

